# Three-dimensional photographic analysis of the face in European adults from southern Spain with normal occlusion: reference anthropometric measurements

**DOI:** 10.1186/s12903-019-0898-y

**Published:** 2019-08-28

**Authors:** M. L. Menéndez López-Mateos, J. Carreño-Carreño, J. C. Palma, J. A. Alarcón, C. Menéndez López-Mateos, M. Menéndez-Núñez

**Affiliations:** 1 Department of Stomatology, Faculty of Odontology, Campus Universitario de Cartuja, University of Granada, 18071 Granada, Spain; 2 Faculty of Odontology, European University, 28670 Madrid, Spain; 3 Department of Stomatology IV, Faculty of Odontology, Complutense University, Plaza de Ramón y Cajal s/n, 28040 Madrid, Spain

**Keywords:** 3D photography, Face, Soft tissues, Anthropometry, Morphometrics, Reference values

## Abstract

**Background:**

Recent non-invasive 3D photography method has been applied to facial analysis, offering numerous advantages in orthodontic. The purpose of this study was to analyze the faces of a sample of healthy European adults from southern Spain with normal occlusion in order to establish reference facial soft tissue anthropometric parameters in this specific geographic-ethnic population, as well as to analyze sexual dimorphism.

**Methods:**

A sample of 100 healthy adult volunteers consisting of 50 women (mean age, 22.92 ± 1.56 years) and 50 men (mean age, 22.37 ± 2.12 years) were enrolled in this study. All participants had normal occlusion, skeletal Class I, mesofacial pattern, and healthy body mass index. Three-dimensional photographs of the faces were captured non-invasively using Planmeca ProMax 3D ProFace^®^. Thirty landmarks related to the face, eyes, nose, and orolabial and chin areas were identified.

**Results:**

Male displayed higher values in all vertical and transversal dimensions, with the exception of the lower lip height. Larger differences between sexes were observed in face, mandible, and nose. Male also had higher values in the angular measurements which referred to the nose. No sex differences were found in transverse upper lip prominence or transverse mandibular prominence. No differences were found in the ratio measurements, with the exception of intercantal width/nasal width, which was higher in women than in men.

**Conclusions:**

Reference anthropometric measurements of facial soft tissues have been established in European adults from southern Spain with normal occlusion. Significant sexual dimorphism was found, with remarkable differences in size between sexes

**Electronic supplementary material:**

The online version of this article (10.1186/s12903-019-0898-y) contains supplementary material, which is available to authorized users.

## Background

Analysis of both hard and soft facial tissues is used in orthodontic diagnoses. Until recently, classical orthodontics considered the study of hard tissues and cephalometric measurements of upper and lower jaws and the teeth as more relevant. These measurements have thus been the most used diagnostic tools in orthodontics [1, 2]. Nevertheless, facial soft tissue morphology has gained increasing interest among clinicians. In fact, currently, orthodontic and maxillofacial surgery diagnoses are not made without the inclusion of specific soft tissue measurements. In addition, lay people (patients and their friends and relatives) asses the success of orthodontic and orthognatic surgery treatments based on perceived visual facial changes [3]. Therefore, a complete three-dimensional (3D) assessment of facial soft tissue shape, size, and proportions should be included as a fundamental step in orthodontic diagnoses, assessment of facial deformities, maxillofacial surgery planning, and evaluation of treatment results [4].

Currently, detailed facial soft tissue examinations can be carried out using 3D radiographic techniques, such as computed tomography, or cone-beam computed tomography (CBCT), which is preferable due to the use of lower radiation doses [5]. Anthropometric facial features can also be analyzed using non-invasive 3D X-ray-free systems, such as laser surface scanning, multi-image photogrammetry, stereo-photogrammetry, or recent 3D facial photography techniques. These new methods offer numerous advantages, including speed of data collection, feasibility of data storage and handling, accuracy, and reliability [6–11].

Reference normative values for specific races and ethnic groups have thus become absolutely necessary [12], as there are remarkable variations between different populations and groups [13]. Some studies have provided reference anthropometric facial data acquired using stereo-photogrammetry or photography from Chinese [4], Korean [14], Malay [15], and Turkish [16] adults. No 3D facial data are available from southern European adult populations

We used a recent non-invasive 3D photography method to analyze the faces of a sample of healthy European adults from southern Spain with normal occlusion. The main aims were to establish standards for facial soft tissue anthropometric parameters in this specific geographic-ethnic population, as well as to analyze sexual dimorphism. We also compared our findings to morphological features of other similarly studied populations.

## Methods

A sample of 100 healthy adult volunteers consisting of 50 women (mean age, 22.92 ± 1.56 years) and 50 men (mean age, 22.37 ± 2.12 years) were enrolled in this study. The inclusion criteria were as follows: 1) European ethnicity, specifically that from Granada in southern Spain. Information regarding ethnicity and geographic origin was obtained using a self-administered questionnaire, which included questions regarding the participants and their parents and grandparents; 2) age between 20 and 30 years; 3) normal occlusion classified as skeletal Class I (based on ANB angle: 0–4°, as measured on a lateral cephalogram), mesofacial growth pattern (according to the Frankfort horizontal-to-mandibular plane angle: 20–28° on a lateral cephalogram), and dental angle Class I; 4) lip competence; and 5) healthy body mass index (18–25 kg/m^2^). The exclusion criteria were 1) craniofacial anomalies; 2) previous or current orthopedic, orthodontic, maxillofacial, or aesthetic surgery treatment; 3) nasal or facial disfigurement, deformity, asymmetry, or surgery; 4) history of facial trauma; and 5) any type of cosmetic facial aesthetic procedure. The sample size was determined using the 3.1.2 version of PS: Power and Sample Size Calculation^®^ according to previously described methods [4, 17].

Participants volunteered for the study after a detailed explanation of the protocol and agreed to participate by signing an ethics committee-approved informed consent form.

Three-dimensional image capture, methods, and measurements

**Fig. 1:**
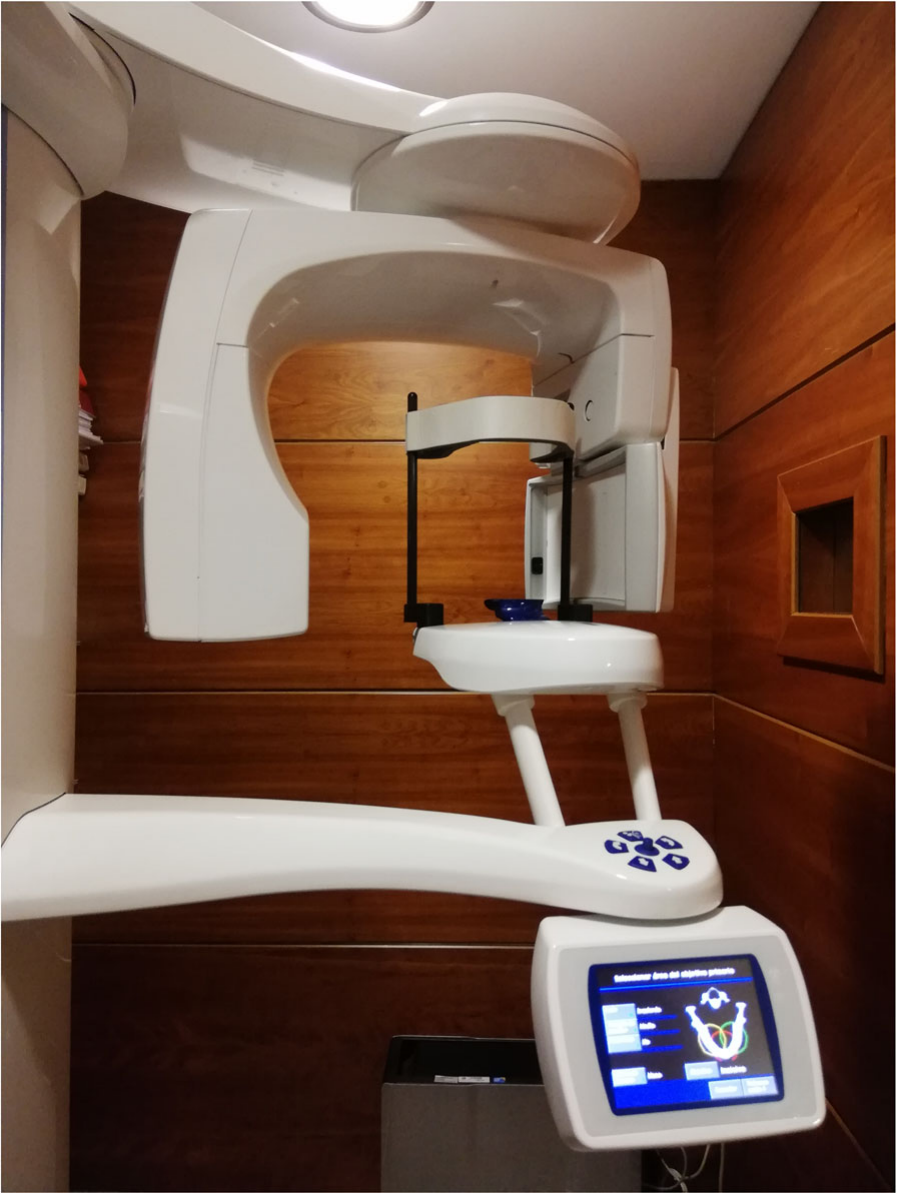
Three-dimensional photography device

Three-dimensional photography of the faces was carried out using Planmeca ProMax 3D ProFace^®^ (Planmeca USA, Inc.; Roselle, IL, USA), which produces a realistic 3D picture of the face (Fig. 1). Photographs were recorded using the ProFace option, which requires no radiation. The system is based on lasers that scan facial geometry and a few digital cameras, which capture texture and color. The sensor components consist of two lights, a laser, two digital cameras, and two light-emitting diodes. The spatial accuracy of this device is 0.03 mm (as reported by the manufacturer). The 3D photographs were processed using Planmeca Romexis^®^ software, which facilitated accurate and detailed operation.

Subjects were instructed not to wear heavy makeup 2 days prior to the scan. They were also instructed to shave and remove their glasses at least 2 h prior to 3D photography. During the image capture, the participant was with the head in a natural position, a neutral facial expression, the mandible in a resting position, and the lips lightly opposed without undue muscular effort.

**Fig. 2:**
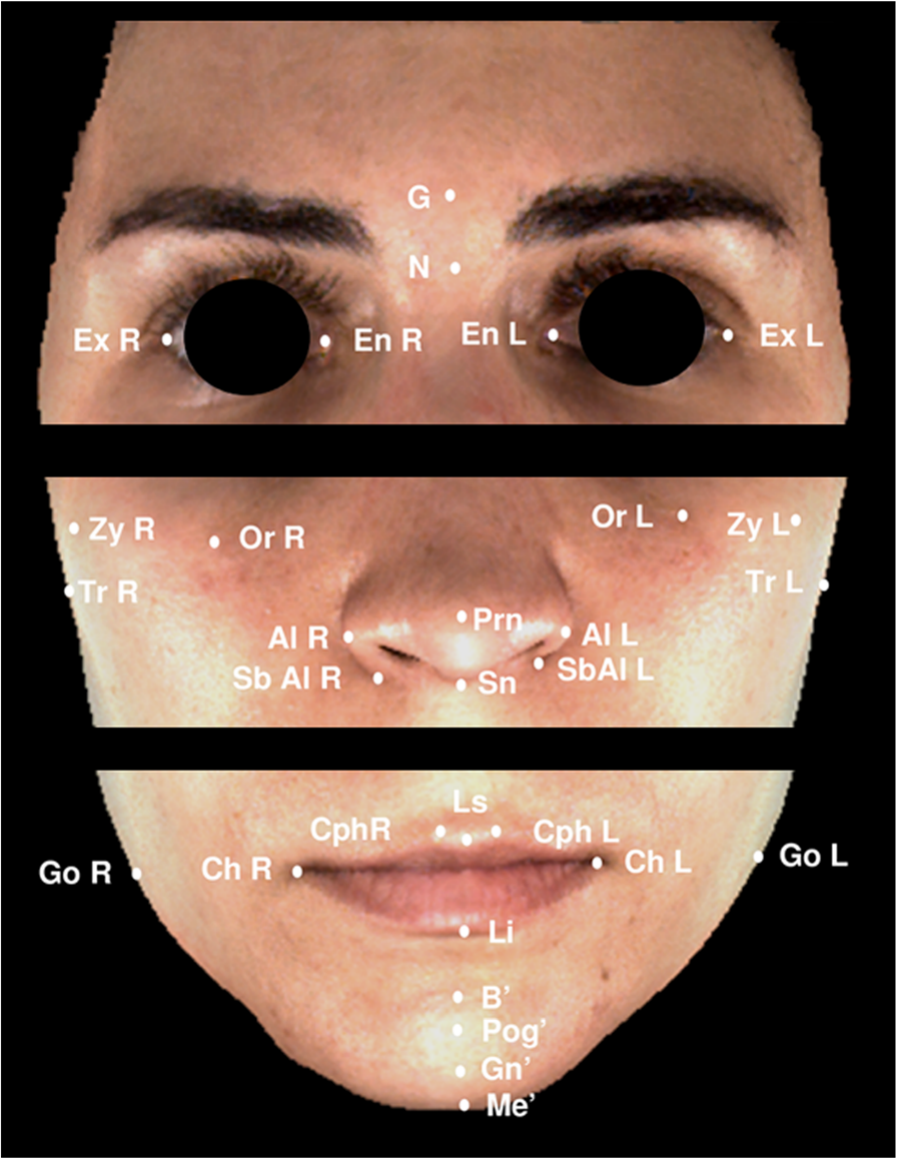
Soft tissue landmarks. N (soft-tissue nasion); G (glabella); Prn (pronasale); Sn (subnasale); Ls (labrale superius); Li (labrale inferius); B (soft-tissue B point); Pg (soft-tissue pogonion); Me (soft-tissue menton); En (endocanthion, R-Right and L-Left); Ex (exocanthion; R-Right and L-Left); Or (orbitale, R-Right and L-Left); Al (alare, R-Right and L-Left); SbAl (subalare, R-Right and L-Left); Cph (christa philtri, R-Right and L-Left) Ch (cheilion, R-Right and L-Left); Zy (zygomatic point, R-Right and L-Left); Go (soft-tissue gonion, R-Right and L-Left); and Tr (tragus, R-Right and L-Left)

Thirty soft tissue anthropometric landmarks related to the face, eyes, nose, and orolabial and chin areas (Fig. 2), based on those suggested by Farkas [18] and Mulliken et al. [19], were identified. The points were recorded manually using Nemotec Arnetts FAB Software^®^, version 10.0 (Software Nemotec SL; Madrid, Spain).

Nineteen linear and 7 angular measurements were used to assess facial anthropometric morphological features, and 12 facial ratios were derived from the linear measurements (Table 1).

**Table 1:** Facial soft tissue anthropometric measurements

Measurements	
Face (mm)	
Face height	N-Me
Lower face height	Sn-Me
Middle facial width	Tr R- TrL
Facial width	ZyR- ZyL
Mandible width	GoR- GoL
Right mandibular body length	GoR-Me
Left mandibular body length	GoL-Me
Nose (mm)	
Nose height	N-Sn
Nasal bridge length	N-Prn
Nasal width	AlR- AlL
Alar base root width	SbAlR- SbAl L
Ocular (mm)	
Biocular width	ExR- ExL
Intercantal width	EnR- EnL
Biorbitale width	OrR- OrL
Orolabial (mm)	
Vermillion height	Ls-Li
Mouth width	ChR-ChL
Philtrum width	CphR-CphL
lower lip height	Li-B
Chin (mm)	
Chin height	Li-Me
	B-Pg
	Pg-Me
Angular measurements (°)	
Nasolabial angle	G-N-Prn
Nasomental angle	N-Prn-Pg
Transverse nasal prominence	Zy R-Prn-ZyL
Transverse upper lip prominence	ChR-Ls-Ch
Transverse mandibular prominence	GoR-Pg-GoL
Ratio measurements	
Upper face height/mandibular width	N-Sn / GoR-GoL
Lower face height/mandibular width	Sn-Me / GoR-GoL
Anterior face height/mandibular width	N-Me/ GoR-GoL
Anterior face height/facial width	N-Me / ZyR-ZyL
Intercantal width/nasal width	EnR-EnL / AlR-AlL
Vermilion height/mouth width	Ls-Li / ChR-ChL
Chin height/right mandibular body length	Li-Me / GoR-Me
Chin height/left mandibular body length	Li-Me / GoL-Me
Nose height/lower face height	N-Sn / SnMe
Nose height/facial width	N-Sn / ZyR-ZyL
Mouth width/intercantal width	ChR-ChL / EnR-EnL
Mandible width/biocular width	GoR-GoL / ExR-ExL

### Statistical analysis

Statistical analyses were performed using Statistical Package for the Social Sciences version 22.0 (Chicago, IL, USA). Descriptive statistics (mean, standard deviation, and standard error of the mean) for each measurement were computed for each sex. Sex differences were tested using Student’s *t* tests. Mann-Whitney U tests were used for measurements with non-normal data. Additional file 1: Table S1 includes all data generated or analyzed during this study (Additional file 1).

Reliability of measurements of the 3D imaging capture system used was tested using the method of moments [20]. Twenty linear measurements were made directly over the faces of 10 randomly selected participants (5 women and 5 men) using an electronic caliper (Ratio^®^). These measurements were then compared to those made indirectly over the 3D images captured from the same participants.

All images were scored by a single experienced observer (MLM). To test for intra-observer reliability, 10 randomly selected images (5 women and 5 men) were scored again after a two-week period. To test for inter-observer reliability, the same 10 randomly selected images were scored by another independent expert (MMN). Inter- and intra-rater agreements were calculated using intra-class correlation coefficients (ICCs).

## Results

The reliability measurements of the 3D images captured by the system indicated a mean reproducibility of 1.04 mm, which is considered adequate for clinical applications [10]. The inter-examiner ICC value was 0.83 (IC 0.61–0.92). The intra-examiner ICC scores ranged from 0.51 (N-Me / ZyR-ZyL) to 0.99 (ChR-ChL / EnR-EnL); the mean ICC score for all of the variables included in the study was 0.84 (IC 0.67–0.99), with 79% of variables having ICC scores > 0.7, which is considered good agreement [21].

Table 2 shows the means, standard deviations, mean differences, and comparisons between male and female subjects for all of the morphological facial variables included in the study. A statistically significant difference was found between male and female subjects in 23 of our 38 measurements. The most prominent differences between the sexes were observed in the measurements obtained from the face region.

**Table 2:** Means, standard deviations, mean differences, and *p*-values for facial morphologic value differences between male and female subjects

Measurements	Male	Female	Mean difference (95% CI)	*p* value
	Mean (SD)	Mean (SD)		
Face (mm)				
Face height	120.40 (8.22)	119.69 (4.25)	3.48 (0.20; 6.75)	0.038*
Lower face height	66.77 (8.05)	65.99 (4.22)	2.77 (−0.46; 6.00)	0.003**
Middle facial width	134.97 (5.44)	128.22 (6.37)	9.04 (6.49; 11.60)	0.000***
Facial width	114.42 (4.63)	110.73 (5.06)	7.39 (4.23; 10.55)	0.000***
Mandible width	113.52 (6.23)	107.58 (7.51)	6.91 (2.80; 11.01)	0.001***
Right mandibular body length	92.56 (13.33)	83.48 (7.45)	7.74 (1.78; 13.70)	0.002**
Left mandibular body length	92.48 (13.96)	83.97 (6.89)	7.12 (1.05; 13.18)	0.008**
Nose (mm)				
Nose height	56.94 (4.45)	56.17 (2.83)	1.58 (0.01; 3.15)	0.049*
Nasal bridge length	48.35 (4.76)	47.56 (2.97)	1.97 (0.04; 3.52)	0.012*
Nasal width	36.62 (3.28)	31.15 (2.21)	5.23 (3.97; 6.50)	0.000***
Alar base root width	23.17 (6.07)	20.17 (3.85)	3.20 (1.25; 5.15)	0.002**
Ocular (mm)				
Biocular width	90.40 (4.68)	86.58 (3.20)	4.83 (3.14; 6.53)	0.000***
Intercantal width	32.52 (4.52)	31.38 (2.78)	2.27 (0.56; 3.98	0.010**
Biorbitale width	76.06 (4.79)	71.97 (4.62)	4.53 (2.60; 6.45)	0.000***
Orolabial (mm)				
Vermilion height	13.07 (3.75)	11.83 (2.45)	1.52 (0.34; 2.70)	0.040*
Mouth width	51.11 (4.77)	47.34 (3.65)	4.21 (2.26; 6.17)	0.000***
Philtrum width	10.62 (2.43)	9.29 (1.95)	1.73 (0.82; 2.64	0.000***
lower lip height	20.25 (3.20)	19.32 (3.69)	1.95 (−1.19; 2.59)	0.695
Chin (mm)				
Chin height	39.07 (7.01)	38.14 (3.68)	2.06 (−0.82; 4.95)	0.004**
B-Pg	6.61 (2.34)	5.69 (1.70)	1.57 (0.37; 2.76)	0.011*
Pg-Me	12.91 (3.22)	12.08 (2.98)	1.77 (0.42; 3.11)	0.011*
Angular measurements (°)				
Nasolabial angle	28.22 (4.32)	24.26 (4.30)	3.92 (1.89; 5.95)	0.000***
Nasomental angle	30.77 (4.00)	28.62 (3.20)	1.94 (0.54; 3.35)	0.000***
Transverse nasal prominence	43.82 (2.08)	41.64 (2.63)	2.76 (1.50; 4.01)	0.000***
Transverse upper lip prominence	35.82 (8.71)	35.53 (3.49)	0.63 (−0.86; 2.08)	0.402
Transverse mandibular prominence	50.62 (3.61)	49.91 (4.20)	1.22 (−0.41; 2.85	0.142
Ratio measurements				
Upper face height/mandibular width	0.50 (0.05)	0,53 (0.05)	−0.02 (− 0.05; 0.00)	0.081
Lower face height/mandibular width	0.59 (0.08)	0.62 (0.06)	−0.01 (− 0.06; 0.03)	0.784
Anterior face height/mandibular width	1,06 (0,09)	1.12 (0.09)	−0.04 (− 0.95; 0.01)	0.140
Anterior face height/facial width	1.05 (0.08)	1.08 (0.05)	−0.03 (− 0.07; 0.00)	0.055
Intercantal width/nasal width	0.89 (0.13)	1.01 (0.11)	− 0.08 (− 0.14; − 0.02)	0.008**
Vermilion height/mouth width	0.26 (0.08)	0.25 (0.06)	0.01 (− 0.01; 0.03)	0.424
Chin height/right mandibular body length	0.42 (0.08)	0.45 (0.07)	−0.02 (− 0.07; 0.02)	0.207
Chin height/left mandibular body length	0.43 (0.09)	0.46 (0.06)	−0.02 (− 0.06; 0.03)	0.332
Nose height/lower face height	0.87 (0.18)	0.86 (0.08)	0.01 (− 0.06; 0.07)	0.175
Nose height/facial width	0.50 (0.05)	0.51 (0.03)	−0.02 (− 0.03; 0.00)	0.063
Mouth width/intercantal width	1.59 (0.21)	1.52 (1.18)	0.01 (−0.11; 0.12)	0.859
Mandible width/biocular width	1.26 (0.08)	1.24 (0.09)	0.15 (−0.03; 0.06)	0.541

*p < 0.05; **p < 0.01; ***p < 0.001

### Face

The male subjects had longer and mostly wider faces than the women. The largest differences were found in the transversal plane, mainly in middle facial width (134.97 ± 5.44 mm in men vs. 128.22 ± 6.37 mm in women) and in facial width (114.42 ± 4.63 mm in men vs. 110.73 ± 5.06 mm in women) (*p* < 0.001). Mandibles were also wider in men than in women, with higher values for mandible width (mean difference, 6.91 mm), and right (mean difference, 7.74 mm) and left (mean difference, 7.12 mm) mandibular body lengths.

### Nose

All measurements (nose height, nasal bridge length, nasal width, and alar base root width) for the nose were significantly larger in men than in women. Larger differences were again found in the transversal dimension (nasal width and alar base root width), with wider noses in men than in women (mean differences, 5.23 and 3.20 mm, respectively).

### Ocular region

The 3 variables used to analyze the ocular region were larger in men than in women. We observed especially large differences in biocular width (90.40 ± 4.68 mm vs. 86.58 ± 3.20 mm) and biorbitale width (76.06 ± 4.79 mm vs. 71.97 ± 4.62 mm).

### Orolabial region

Vermilion height, mouth width, and philtrum width were significantly larger in men than in women. No statistically significant sex difference was found in lower lip height.

### Chin

All evaluated chin measurements were significantly larger in men than in women, with a large difference in chin height (39.07 ± 7.01 mm vs. 38.14 ± 3.68 mm).

### Angular measurements

Significant sex differences in angular measurements were found in the nose region (nasolabial angle, nasomental angle, and transverse nasal prominence) (*p* < 0.001). Transverse upper lip prominence and transverse mandibular prominence were similar in both sexes.

### Ratio measurements

We found no significant sex differences in ratio measurements, with the exception of the intercantal width to nasal width ratio, which was higher in women than in men (*p* < 0.01).

## Discussion

In spite of the recent increase in the relevance of soft tissue facial analysis, there is an absence of reference values for some races, ethnicities, and geographic population groups. These data are required to determine deviations from standard measurements. We used a recent non-invasive 3D photography method to analyze the faces of a sample of healthy European adults with normal occlusion from southern Spain. We established anthropometric facial soft tissue reference values for this specific geographic-ethnic population. We also investigated differences between the sexes in this population.

We found clear sexual dimorphism, with statistically significant differences between male and female subjects in most facial variables that were analyzed. The male subjects had higher values in all vertical and transversal dimensions, with the exception of lower lip height, which was similar in the two groups. The male subjects also had higher values in the angular measurements of the nose. No sex differences were found in transverse upper lip prominence or transverse mandibular prominence. Only one statistically significant sex difference was found in the ratio measurements (intercantal width/nasal width, which was higher in women than in men). The rest of the measured ratios were similar in both sexes.

Planmeca ProFace™, which was used to capture facial soft tissue characteristics, generates 3D photos in one imaging session while the patient position, facial expression, and muscle position remain unchanged. This leads to the production of images that are perfectly compatible (technical information provided on the company website) (http://www.planmeca.com/Imaging/3D-imaging/Planmeca-ProFace/). The reliability of the measurements produced by the 3D imaging capture system used was tested using the method of moments. Specifically, we compared the direct measurements (those made over the face of the patient using an electronic caliper) with the same measurements made indirectly (over the 3D images captured using Planmeca ProMax 3D ProFace^®^ [Planmeca USA, Inc.; Roselle, IL, USA]) using the same randomly selected participants. The results indicated adequate reproducibility (mean, 1.04 mm) [10].

The 3D photography method offers many advantages over conventional (non-3D) photography, including accurate 3D images and reliability to perform facial analysis. Nevertheless, a more sophisticated device and software are required.

In our population, which consisted of European adults from southern Spain, prominent sex differences were observed in measurements of the face, mandible, and nose. These measurements were significantly larger in men than in women.

In our study, the male subjects had longer and wider faces than the female subjects. Similar results were found by Baik et al. in Korean adults [14] and by Ozdemir et al. in Turkish young adults [16]. Othman et al. also described longer faces in men than in women in a Malaysian population, although they did not include facial width measurements [15]. There are also differences between populations: Korean men have slightly longer faces than Europeans from southern Spain (face height, N-Me, 121.42 ± 6.03 mm vs. 120.40 ± 8.22 mm), while women from southern Spain have longer faces than Korean women (119.69 ± 69 mm vs. 114.41 ± 5.89 mm). Sexual dimorphism in face height was more prominent in the Korean population [14]. Our results are not comparable with those obtained in Turkish [16] or Malaysian [15] populations. This is because, in those studies, the authors considered face height as the distance from N to Gn, although they also found higher sexual dimorphism than we did. Sexual dimorphism has also been reported in a Chinese population [4], although the different methodology used makes it difficult to compare the Chinese study to ours.

Mandible width and right and left mandibular body length were also significantly larger in men than in women in our southern European sample. Similar results were found in Turkish and Korean adults, with wider mandibles in men than in women. Inter-group differences can be observed when comparing populations: Koreans men and women have the widest mandibles (measured from right to left gonion) (127.38 ± 7.43 mm in men and 118.01 ± 7.41 mm in women). They are followed by the Turkish (116.3 ± 1.26 mm in men and 110.2 ± 1.65 mm in women) and the southern Europeans in our study, who had the smallest mandible width (113.52 ± 6.23 mm in men and 107.58 ± 7.51 mm in women). These inter and intra-population differences in face and mandible size and shape may be attributed to several factors, including genetic or environmental factors, as suggested by paleo-anthropology studies [22–26].

In our study, all the measurements of the nose had larger values in men than in women. This was especially true of nasal width, which had a mean difference of 5.23 mm. Sexual dimorphism in nose dimensions had also been described in Malaysian adults. Malaysian men have generally longer and more prominent noses. In addition, nose height and nasal bridge length are significantly in Malaysian men (mean differences of 4.93 mm and 5.73 mm, respectively) [15]. Baik et al. [14] also found longer and more prominent noses in men than in women. In contrast, Ozdemir et al. [16] did not find sexual dimorphism in the height of the nose, the length of the nasal bridge, or the nasal root width in Turkish adults. Our southern European population had narrower noses (nasal width: 36.62 ± 3.28 mm in men and 31.15 ± 2.21 mm in women) than other racial and ethnic groups [14–16, 27–29]. In contrast, the nose height was had higher values in our group (56.94 ± 4.45 mm in men and 56.17 ± 2.83 mm in women) than in Malaysian (54.13 ± 3.61 mm in men and 49.20 mm in women) [15], Chinese (50.15 ± 4.16 mm in men and 46.93 ± 3.3 mm in women) [30], Turkish (51.9 ± 0.75 mm in men and 51.7 ± 0.58 mm in women) [16], and Korean (53.26 ± 3.46 mm in men and 48.4 ± 4.52 mm in women) [14] populations. Our results were similar to those found in white northern Italians (57.43 ± 3.93 mm in men and 54.07 ± 3.68 mm in women) [27]. A proposed explanation for sexual differences in nose dimensions is that men have higher daily energy expenditure, greater respiratory air consumption, and different body composition [22, 31].

Sexual dimorphism was also found in the ocular region in our population, with significantly higher values in men than in women for all variables analyzed. Major differences were found in biocular width (mean difference, 4.83 mm) and biorbitale width (mean difference, 4.53 mm). In the study by Othman et al. [15], only biocular width (mean difference, 4.14 mm) was significantly larger in Malay men than in Malay women. Although the main differences in biocular width were quite similar in both studies, the Malaysian subjects had higher values for both men (96.19 ± 4.64 mm) and women (92.05 ± 3.22 mm) than those found in our southern European sample (90.40 ± 4.68 mm in men and 86.58 ± 3.20 mm in women). No sexual dimorphism was found in a Korean population [14], although the linear distance ExR-ExL (what is referred to as ‘upper face width’) was even higher (106.75 ± 6.13 mm in men and 104.98 ± 5.47 mm in women) in that population. There are thus large differences in the ocular area between races and ethnic groups.

All of the measurements in the orolabial were significantly larger in men than in women, with the remarkable exception of lower lip height, which did not display a significant sex difference. Similarly, no sexual dimorphism was found in the lower vermilion height in a Turkish population [16]. In our sample, philtrum width (10.62 ± 2.43 mm in men and 9.29 ± 1.95 mm in women) and mouth width (51.11 ± 4.77 mm in men and 47.34 ± 3.65 mm in women) values were similar to those found by Othman et al. [15] in Malaysians (11.84 ± 1.90 mm in men and 10.40 ± 1.14 mm in women, and 50.83 ± 3.75 mm in men and 48.00 ± 2.61 mm in women, respectively). The above authors, however, found smaller differences in mouth width between sexes (2.83 mm vs. 4.21 mm in our study). Turkish [16] and Korean [14] subjects have wider philtrums in both sexes. The widest mouths are found in white northern Italians [27] (55.71 ± 3.81 mm in men and 50.84 ± 3.83 mm in women). The narrowest mouths are found in Turks [16] (47.1 ± 0.54 mm in men and 44 ± 0.31 mm in women).

Angular and ratio measurements are difficult to compare among the published studies due to the different methodologies and variables that have been considered. In our population, nasolabial, nasomental, and transverse nasal prominence angles were significantly larger in men than in women. In contrast, transverse upper lip prominence and transverse mandibular prominence did not show sexual dimorphism. Thus, there were significant sex differences in the angular measurements of the nose between the sexes. Baik et al. [14] also did not find significant sex differences in angular measurements, with the exception of the nasal frontal angle and the transverse nasal prominence, in a Korean population. Othman et al. [15] did not find clinically significant differences between the sexes in angular and ratio measurements in Malaysians.

Among the ratio measurements in our study, only the intercantal width/nasal width ratio was significantly different between the sexes, with higher ratios in women (1.01 ± 0.11) than men (0.89 ± 0.13). In contrast, Baik et al. [14] found significant sex differences in the ratio of anterior facial height to the interzygomatic distance and that of forehead height to forehead width. However, the ratio of facial height to upper facial height relative to mandibular width was similar in both sexes, which is consistent with our study. These results suggest that there are larger differences in the sizes, rather than the shapes, of faces between men and women. The comparisons and differences with other populations reported in our study should be interpreted with caution due to the different systems used for facial evaluation, as well as in the different variables used to analyze anthropometric facial features.

## Conclusions

Here we establish reference anthropometric measurements of facial soft tissues in European adults from southern Spain with normal occlusion using non-invasive 3D photography. Most of the parameters had significant sexual dimorphism. Men had higher values in all vertical and transversal dimensions, with the exception of lower lip height, which was similar in the two groups. The greatest differences between sexes were observed in measurements obtained from the face, mandible, and nose, which were significantly larger in men than in women. However, only one statistically significant sex difference was found in the ratio measurements (intercantal width/nasal width, which was higher in women than in men).

## Additional file

Additional file 1: Variables data generated or analyzed during this study. (XLSX 51 kb)

## Data Availability

All data generated or analysed during this study are included in this published article and its supplementary information files.

## Abbreviations

3D: Three dimensional
Al: Alare
B: B point
CBCT: Cone-beam computed tomography
Ch: Cheilion
Cph: Christa philtri
En: Endocanthion
Ex: Exocanthion
G: Glabela
Go: Gonion
ICCs: Intra-class correlation coefficients
L: Left
Li: Labrale inferious
Ls: Labrale superious
Me: Menton
N: Nasion
Or: Orbitale
Pg: Pogonion
Prn: Pronasale
R: Right
SbAl: Subalare
Sn: Subnasale
Tr: Tragus
Zy: Zygomatic point
